# Data protection and fairness by design

**DOI:** 10.1093/eurpub/ckac129.083

**Published:** 2022-10-25

**Authors:** J Rachadell

**Affiliations:** 1 Public Health Unit, ACES Lisboa Ocidental e Oeiras, Lisbon, Portugal; 2 Institute for Evidence Based Health, Lisbon, Portugal; 3 EUPHA-DH

## Abstract

Building a health data pipeline is an opportunity not only to review the efficiency of data flows and organisational processes but also to guarantee that the resulting data system incorporates data protection and fairness principles by design. This session will cover some key aspects of data protection and the principles of data fairness and ethics:

Transparency, simplicity and fairness

The importance of a diverse team with diverse expertise

Keeping the business objectives in mind and purpose limitation

Compliance with data protection directives (GDPR)

